# Drug Repositioning as a Therapeutic Strategy against *Streptococcus pneumoniae*: Cell Membrane as Potential Target

**DOI:** 10.3390/ijms24065831

**Published:** 2023-03-18

**Authors:** Laura Ortiz-Miravalles, Manuel Sánchez-Angulo, Jesús M. Sanz, Beatriz Maestro

**Affiliations:** 1 Protein Engineering against Antimicrobial Resistance Group, Centro de Investigaciones Biológicas Margarita Salas, Consejo Superior de Investigaciones Científicas (CSIC), 28040 Madrid, Spain; lortiz05@ucm.es; 2 Department of Animal Health, Faculty of Veterinary Medicine, Universidad Complutense de Madrid, 28040 Madrid, Spain; 3 VISAVET Health Surveillance Centre, Universidad Complutense de Madrid, 28040 Madrid, Spain; 4 Department of Vegetal Production and Microbiology, Universidad Miguel Hernández, 03202 Elche, Spain; m.sanchez@umh.es; 5 Centro de Investigación Biomédica en Red de Enfermedades Respiratorias (CIBERES), Instituto de Salud Carlos III, 28029 Madrid, Spain; 6 Department of Biochemistry and Molecular Biology, Faculty of Biology, Universidad Complutense de Madrid (UCM), 28040 Madrid, Spain

**Keywords:** pneumococcus, antimicrobial resistance, drug repurposing, membrane permeability

## Abstract

A collection of repurposing drugs (Prestwick Chemical Library) containing 1200 compounds was screened to investigate the drugs’ antimicrobial effects against planktonic cultures of the respiratory pathogen *Streptococcus pneumoniae*. After four discrimination rounds, a set of seven compounds was finally selected, namely (i) clofilium tosylate; (ii) vanoxerine; (iii) mitoxantrone dihydrochloride; (iv) amiodarone hydrochloride; (v) tamoxifen citrate; (vi) terfenadine; and (vii) clomiphene citrate (Z, E). These molecules arrested pneumococcal growth in a liquid medium and induced a decrease in bacterial viability between 90.0% and 99.9% at 25 µM concentration, with minimal inhibitory concentrations (MICs) also in the micromolar range. Moreover, all compounds but mitoxantrone caused a remarkable increase in the permeability of the bacterial membrane and share a common, minimal chemical structure consisting of an aliphatic amine linked to a phenyl moiety via a short carbon/oxygen linker. These results open new possibilities to tackle pneumococcal disease through drug repositioning and provide clues for the design of novel membrane-targeted antimicrobials with a related chemical structure.

## 1. Introduction

Antimicrobial resistance (AMR) constitutes an ever-growing, first-order global problem that has been identified as a One Health challenge, affecting people, animals, and the environment [1,2]. Antibiotic resistance makes the infections difficult and sometimes impossible to treat: In 2019, the global burden associated with drug-resistant infections across 88 pathogen–drug combinations was estimated to be 4.95 million deaths, 1.27 million of which would be directly attributable to AMR [1,2]. In this context, the research and development of new drugs against the most threatening healthcare pathogens are among the key actions to which both the World Health Organization (WHO) and the US Centers for Disease Control and Prevention (CDC) have paid attention [1,2].

One of the major pathogens worldwide affected by AMR issues is *Streptococcus pneumoniae* (pneumococcus) [2,3]. It is a major causative agent of pneumonia, meningitis, sepsis, and otitis media, with 1 million deaths estimated per year [4], and it is the leading organism in terms of years of life lost (YLL), with 40.3 million YLL [5]. Although antibiotic-resistant invasive pneumococcal infections have decreased since the introduction of polysaccharide conjugate vaccines, more than 2 million pneumococcal infections occur every year only in the United States, with more than 30% resistant cases to one or more clinically relevant antibiotics, resulting in more than 6000 deaths and USD 4 billion in total costs [2]. Furthermore, in the present situation of the COVID-19 pandemic, the participation of *S. pneumoniae* as the major co-infecting agent with SARS-CoV2 has been thoroughly documented [6], while some recent results also suggest that invasive pneumococcal disease may increase the predisposition to undergo this viral infection and aggravate the illness [7].

The lack of effective antimicrobials for the treatment of certain pneumococcal strains has made it a priority pathogen for the investigation of new therapeutic strategies [2,8] even more, as in recent years, the clinical pipeline of new antimicrobials is drying, and few new antibiotics have been approved by the US Food and Drug Administration [5]. In our search for novel antipneumococcal molecules, we have described the antimicrobial effects of esters of bicyclic amines (EBAs), a set of membrane-perturbing compounds with broad bactericidal effects that are especially effective on *S. pneumoniae* due to their ability to prematurely release the major autolysin of the pathogen from its cytoplasmic pool [9]. On the other hand, an attractive strategy to combat AMR is drug repositioning, based on pharmaceutical agents already approved by regulatory agencies for the treatment of new pathologies, leading to the fast deployment of drugs with novel activities that could rapidly provide benefits to patients while reducing healthcare costs [10,11]. In this sense, the Prestwick^®^ Chemical Library (1200 compounds in the version used in this work) comprises a collection of off-patent, approved drugs, either by the US Food and Drug Administration (FDA), the European Medicines Evaluation Agency, or other regulatory institutions, with diverse mechanisms of action and well-known pharmacological and toxicological properties in humans. This library has been widely and successfully screened to identify potential repositionable drugs [12,13,14,15]. The wide chemical diversity of this library makes it likely to find compounds with similar antipneumococcal mechanisms as the EBAs that are therefore amenable to displaying unprecedented activity against this pathogen. Accordingly, the goal of this work was the identification of molecules with in vitro activity against *S. pneumoniae* not previously described and that could be subjected to repurposing strategies in the future.

## 2. Results

### 2.1. Library Screening

**Figure 1:**
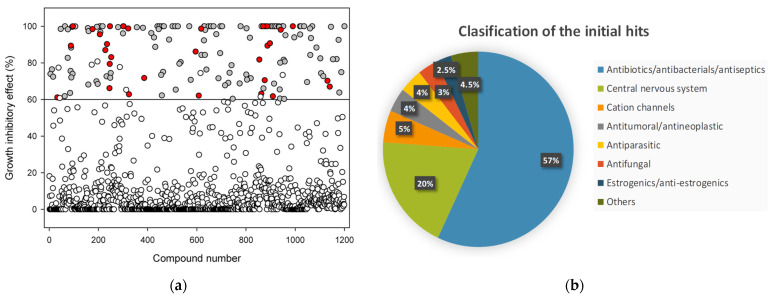
Results of the screening of the Prestwick Chemical Library on pneumococcal planktonic growth: (**a**) Decrease in the OD_550_ of the culture induced by the presence of 50 μM of each compound after 4 h (mid-stationary phase), expressed as mean percentage of inhibitory effect relative to the 0.5% DMSO control curve. Compounds inducing a decrease in optical density higher than 60% relative to the control in the stationary phase are represented as all circles above the horizontal line (161 drugs); white circles above the line represent those compounds that could not prevent the optical density of the culture to reach more than 50% relative to the control at any other growth phase (9 drugs) and were subsequently discarded, leading to 152 initial hits (grey and red circles); among these, grey circles represent those that correspond to already described antistreptococcal drugs (121 drugs) and were also discarded for further analysis; finally, red circles represent the 31 remainder compounds that were subjected to further screening rounds; (**b**) composition of initial hits (152 compounds, red, and grey circles in panel (**a**), according to their pharmacological properties.

The first screening round was performed in a blind assay on pneumococcal planktonic cultures grown in a C+Y medium in multiwell plates containing each of the 1200 compounds at 50 µM. Samples contained 0.5% dimethyl sulfoxide (DMSO) as the final concentration after dilution from the stock. To ensure the homogeneous distribution of bacteria in the well, we employed the non-capsulated strain R6CIB17, a variant of the R6 laboratory strain that does not flocculate in a liquid medium [16]. Each compound was added to the culture at the early exponential phase (OD_550_ ≈ 0.15), and the growth was monitored via turbidimetry. DMSO at the mentioned residual concentration only showed minor effects as compared with the control (Supplementary Material Figure S1). We then first selected as positively scoring molecules (hits) those that, 4 h after compound addition (i.e., in the mid-stationary phase (Supplementary Material Figure S1)), displayed a decrease in the OD_550_ higher than 60% relative to the control with DMSO (Figure 1a, all circles above horizontal line, 161 drugs). After this, we applied a second, more stringent filter to discard those compounds that were unable to prevent the maximum OD_550_ of the culture to reach more than 50% of the control at any other growth phase (Figure 1a, white circles above the horizontal line). This screening resulted in a total of 152 initial hits (Figure 1a, grey and red circles above the horizontal line).

Most of the initial hits (86/152, 57%) correspond to antibacterial drugs, including well-known beta-lactam, tetracycline, or fluoroquinolone antibiotics (Figure 1b), and all of them previously identified as antipneumococcal drugs, which on the other hand, confirmed the validity of the screening technique. Among the remaining hits, the most represented set corresponds to central nervous system drugs (30/152, 20%). In a lower proportion, the screening yielded compounds with different ion-channel-associated activities (8/152, 5%), antimalarial/antiparasitic drugs (6/158, 4%), anticancer/antineoplastic effectors (6/151, 4%), antifungals (5/152, 3%), and estrogenic or antiestrogenic compounds (4/152, 3%). Out of the 152 candidates, 121 corresponded to compounds with already described antistreptococcal activity. Therefore, 31 drugs were selected that, while to our knowledge, had not previously been reported showing any activity against pneumococcus, they appreciably affected or completely inhibited the bacterial growth in the liquid medium relative to the control at 50 μM concentration (Figure 1a, red circles; Supplementary Material Figure S1; Supplementary Material Table S1).

**Figure 2:**
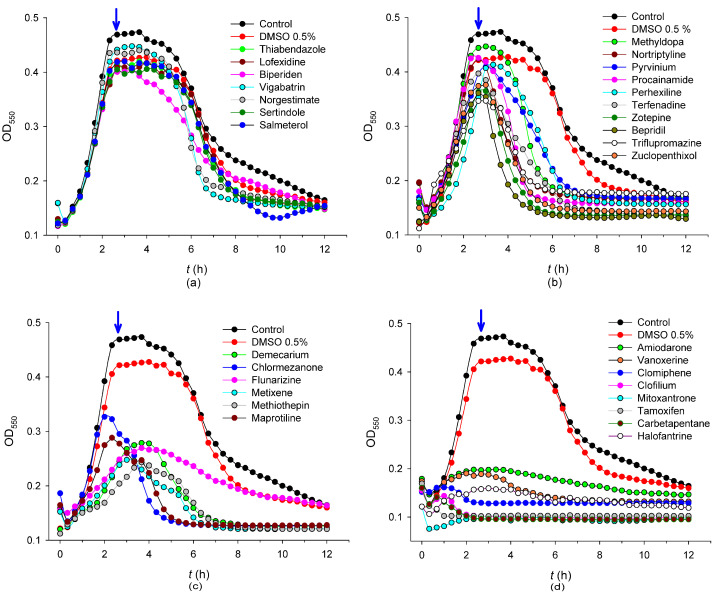
Effect of 31 selected compounds (25 µM) (Table 1) on pneumococcal planktonic growth. Arrows indicate the time of removal of samples for the viability assays (Table 1): (**a**) no significant effect; (**b**) weak effect; (**c**) notable effect; (**d**) strong effect.

**Figure 3:**
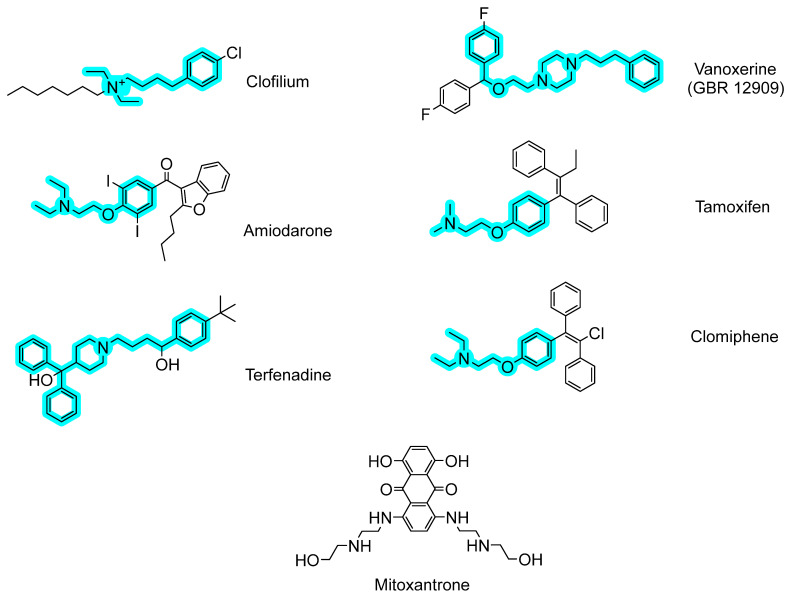
Structures of compounds affecting pneumococcal viability in more than 90%. The fragment structure common to all molecules except mitoxantrone is highlighted in cyan.

Next, as a third selection round, we evaluated the effect of this set on the pneumococcal growth at 0.1, 1, 5, and 25 µM concentrations, but we only observed appreciable effects in 24 drugs at the highest concentration (Figure 2b–d and Table 1), which were deemed as possible repurposing candidates on which to focus our investigation. To determine the effect of the compounds on the bacterial viability at 25 µM, samples of the planktonic cultures were taken at the onset of the stationary phase (140 min after compound addition) (Figure 2 and Table 1). Among the 24 aforementioned hits, we identified 7 compounds that induced a decrease in bacterial viability between 90.0% and 99.9% relative to the control (DMSO 0.5%), namely clofilium tosylate, vanoxerine (GBR 12909 dihydrochloride), mitoxantrone dihydrochloride, amiodarone hydrochloride, tamoxifen citrate, terfenadine, and clomiphene citrate (Z, E) (Figure 3 and Table 1). All of these compounds except terfenadine severely impaired growth in planktonic cultures throughout all growth phases (Figure 2d). The effect of terfenadine on the growth was only evident at the end of the exponential phase (Figure 2b), which may be indicative of a slow bactericidal response that is only unveiled in long-term viability assays (Table 1). On the other hand, although both carbetapentane citrate and halofantrine hydrochloride remarkably impaired the planktonic growth (Figure 2d), they only displayed a modest effect on viability (Table 1), and for this reason, they were not included in further analysis. This phenomenon may in any case reflect a bacteriostatic mechanism exerted by these two compounds.

Finally, we determined the minimal inhibitory concentration (MIC) of the seven most promising bactericidal drugs in both the non-virulent, non-capsulated (R6) strains and capsulated (D39) strains using the Clinical and Laboratory Standards Institute (CLSI) standard and with a microdilution system in plates. Concentrations ranged from 12 to 51 µg mL^−1^ in the R6 strain and from 26 to 56 µg mL^−1^ in the capsulated D39 strain (Table 1). The compound with the lowest MIC on D39 was vanoxerine (26 µg mL^−1^). On the other hand, despite their clear effect on cell viability (Table 1), the MIC values for mitoxantrone and tamoxifen were too high to be measured within the range of our experimental conditions (Table 1).

### 2.2. Antibacterial Mechanisms of the Selected Hits: Membrane Destabilization Studies

The chemical structure of the seven final hits selected in the screening is depicted in Figure 3. With the exception of mitoxantrone, the structures of all compounds show several common traits, namely (i) a tertiary/quaternary aliphatic amine with ethyl or longer aliphatic substituents (methyl in the case of tamoxifen); (ii) a phenyl ring not belonging to a polycyclic system, without polar substituents and connected to the amine nitrogen through single-bonded 4–5 carbon/oxygen atoms; and (iii) a high value of the cLogP parameter (Table 1), denoting a relevant hydrophobicity.

**Figure 4:**
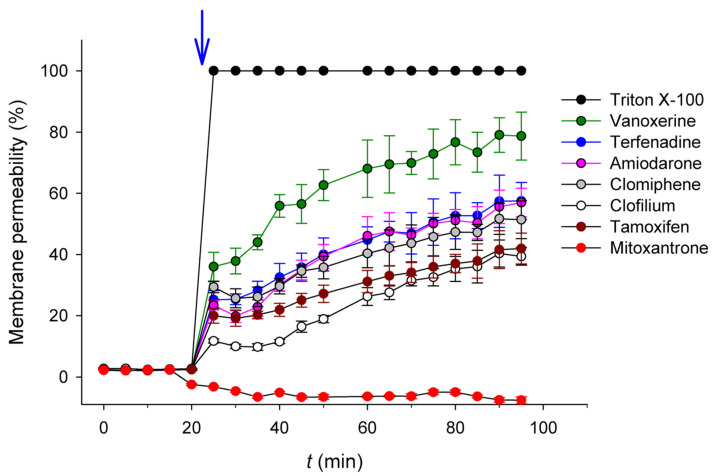
Evaluation of the membrane permeability of the pneumococcal cells upon the addition of compounds (25 µM). The arrow indicates the time of addition.

Figure 3 also highlights in cyan this minimal fragment structure shared by the mentioned compounds. It is remarkable that these characteristics were not found in any of the rest of the 24 compounds selected in the previous round (see Table 1). The fact that the amine group of these molecules would be positively charged at physiological pH, together with the hydrophobicity of their linked moieties, prompted us to speculate that these amphiphilic compounds might present a common “off-target” mechanism consisting of the perturbation of the pneumococcal cell membrane through the ionic interaction with the negatively charged polar head of phospholipids, together with the interaction of the hydrophobic moiety with fatty acid chains [9,17,18,19,20]. To check this hypothesis, the effect of the selected hits on the cell membrane of *S. pneumoniae* was analyzed by monitoring the entrance of exogenously added SYTOX Green as a fluorescent probe. SYTOX Green is a nucleic acid stain molecule that is not able to cross the cell membrane unless it is depolarized by a membrane-active agent, allowing its binding to DNA and increasing its fluorescence emission signal by about 500-fold [21]. Triton X-100 was used as a positive control for 100% permeability. Figure 4 shows that as early as 5 min after the addition of the selected compounds at 25 µM concentration, bacterial cells started showing a steady and significant increase in the fluorescence signal in all cases, with the exception of mitoxantrone, and vanoxerine was the most effective (approx. 80% of maximum permeability after 90 min, see Table 1). These results indicate that all compounds but mitoxantrone were able to destabilize the *S. pneumoniae* membrane.

## 3. Discussion

Multidrug resistance and its fast rate of dissemination are major problems of public health, resulting in considerable morbidity, mortality, and healthcare costs [22,23]. *S. pneumoniae* constitutes a clinically important, primary pathogen responsible for pneumonia, meningitis, or acute otitis media, and multidrug- and extremely drug-resistant strains are very difficult or unfeasible to treat [22,23,24]. This situation has been aggravated by the slow pace of new antimicrobial drug development, which has led both the WHO and CDC to appoint pneumococcus as one of the pathogens for which there is an urgent need for research and development of alternative strategies and new antimicrobials [22,23].

In this context, we screened 1200 commercial drugs contained in the Prestwick Chemical Library on their negative effect on planktonic pneumococcal growth and viability. The application of four stringent selection rules resulted in the final identification of seven molecules (Table 1 and Figure 3) with no antipneumococcal properties previously reported, which are firm candidates either as repurposed antimicrobial drugs or as lead compounds for further development, as they induced a decrease between 90% and 99.9% in bacterial viability at 25 µM concentration, and their MIC values overall fell within the range of other known antimicrobials such as clarithromycin, clindamycin, or erythromycin [25,26,27] (Table 1). Except for mitoxantrone, the rest of the molecules induced a notable increase in cell membrane permeability (Figure 4 and Table 1), which may be the underlying cause of their bactericidal effect. In fact, vanoxerine is the molecule causing a higher perturbation of the membrane (Figure 4) and is also the compound with the lowest MIC (Table 1). In this regard, all the active molecules but mitoxantrone share a common, unique chemical fragment core structure distinctive from the rest of the hits (Figure 3), in particular, a positively charged aliphatic tertiary/quaternary amine attached to an aromatic, lipophilic moiety through a linear carbon/oxygen linker, leading to an amphiphilic structure that might explain their bactericidal activity by selectively altering or disrupting the bacterial membrane permeability, followed by the lysis of the pneumococci. In support of this idea, amiodarone has been shown to be effective against many other Gram-positive and Gram-negative bacteria [28] by perturbing the phospholipid bilayer structure [29], a mechanism that has also been suggested for tamoxifen on the non-pathogenic *Bacillus stearothermophilus* [30]. Moreover, clofilium tosylate shows activity against *Acinetobacter baumannii* and a more potent effect against *Staphylococcus aureus* by inducing non-specific membrane permeability [31,32]. In addition, these highly lipophilic compounds may partition into hydrophobic membranes and inhibit numerous cellular functions mediated by membrane proteins or collapse the proton motive force. In this sense, amiodarone, clomiphene, and tamoxifen have been shown to be uncouplers [33], and in the case of clomiphene, it has also been described to be an *S. aureus* growth inhibitor by targeting the cell-wall biosynthesis through the inhibition of undecaprenyl diphosphate synthase [33]. Nevertheless, other structural features may also play a relevant role in bactericidal activity. For instance, both tamoxifen and clomiphene, an estrogen receptor antagonist and an antiestrogen gonad-stimulated agent, respectively, present a triphenylethylene backbone, which has been claimed as a biologically privileged scaffold for the treatment of infectious diseases [34].

Antibacterial strategies based on non-specific membrane distortion, either by inducing direct physical changes and/or by disturbing embedded targets, impede the ability of bacteria to acquire resistance mechanisms and are therefore attractive procedures to control infection [35]. In this sense, the physical–chemical properties of the selected drugs may provide structural clues for the rational design of novel antipneumococcal molecules aimed at membrane perturbation. For instance, the selected compounds display at least ethyl-long aliphatic substituents in the nitrogen atom, except tamoxifen, which only contains two shorter methyl groups, and which, remarkably, together with the distinctive mitoxantrone (see below), is the only compound with a MIC exceeding the limits of our assay (Table 1). On the other hand, the overall structure of this group is also reminiscent of that of the esters of bicyclic amines (EBAs), such as atropine and ipratropium, a family of compounds that have already been shown to induce the autolysis of pneumococcal planktonic cultures and biofilms through the destabilization of the membrane that leads to the premature release of the major LytA autolysin from its cytoplasmic pool, finally causing massive cell-wall degradation [9,36].

However, alternative mechanisms of action of these compounds besides the perturbation of the membrane cannot be ruled out. For instance, the antihistamine and channel blocker terfenadine displays antimicrobial activity on both planktonic and biofilm cultures of *S. aureus* and other Gram-positive bacteria by inhibiting the DNA gyrase and topoisomerase IV [37]. Moreover, vanoxerine has also been described as affecting the 3-dehydroquinote synthase, an enzyme involved in the biosynthesis of aromatic amino acids in *Mycobacterium tuberculosis* [38] that displays a high sequence similarity with the pneumococcal protein (). On the other hand, it is noteworthy that half of the seven selected compounds (vanoxerine, clofilium, amiodarone, and terfenadine) are ion channel blockers. Such a weighty representation of this group of drugs is in accordance with some reports of ion channel effectors as active antimicrobial compounds by inhibiting bacterial efflux pumps [39] and points to further, specific screenings using libraries with a more ample representation of this group of drugs. Nevertheless, it should be noted that not all of the channel blockers contained in the library resulted to be effective against pneumococci, even though some of them have been described as antimicrobials, such as felodipine [40,41], again suggesting that chemical constraints may also be necessary to determine to become effective antipneumococcal drugs.

Mitoxantrone represents a particular case that deviates from the rest of the hits. It did not affect the integrity of the pneumococcal cell membrane (Figure 4), its structure does not share any common trait with the other compounds (Figure 3), and its MIC was too high to be accurately measured (Table 1). This suggests a distinctive antibacterial mechanism definitively other than acting on the membrane. Mitoxantrone is a synthetic anthracenedione, therefore bearing the anthracycline structural core that is presently used as an anticancer agent in hospitals [42]. Together with other anthraquinones, it has also attracted interest due to its broad-spectrum antibacterial activity, thus receiving consideration as an antineoplastic antibiotic [43,44,45,46,47]. Nevertheless, mitoxantrone is less cardiotoxic than other anthracycline antineoplastics such as adriamycin and daunomycin [48], and many compounds having anthraquinone scaffolds have been synthesized and proved effective over a wide range of Gram-positive and Gram-negative bacteria with lower cytotoxic effects [49]. For instance, the planar, the hydrophobic anthraquinone structure of amsacrine (m-AMSA), facilitates its intercalation in the double DNA helix with the subsequent inhibition of the gene-39 subunit of the *Escherichia coli* T4 bacteriophage-encoded type-II DNA topoisomerase [50], a protein with high similarity with the B subunit of type-II topoisomerase IV from *S. pneumoniae*. In this line, DNA supercoiling machinery might constitute another promising target for the discovery of novel antipneumococcal drugs, as it has been described that targeting the pneumococcal type-I topoisomerase I with the planar seconeolitsine molecule exerts an in vivo protection against invasive pneumococcal disease [51].

The antimicrobial effectivity of the selected compounds identified in this work hints at a promising potential that deserves to be further explored upon designing the corresponding in vivo experiments. All these drugs have protein or DNA receptors as targets in humans [38,52,53,54,55,56], so the interaction with the bacterial membrane seems to be quite specific to the prokaryotic organisms and therefore side effects derived from binding to the eukaryotic membrane seem unlikely (at least at the concentrations tested). Among all the selected compounds, vanoxerine could be considered one of the most promising ones. It is the molecule with the highest permeability activity and lowest MIC for the capsuled D39 strain (Figure 4 and Table 1) and an investigational drug not yet approved for therapeutic use but that has already completed a phase-III trial for atrial fibrillation or flutter [57]. Furthermore, vanoxerine is able to cross the blood–brain barrier [58], making it especially apt to treat pneumococcal meningitis. Another attractive candidate is clomiphene, a drug that is readily absorbed, shows no reported toxic effects upon acute use, and has tolerable side effects [56]. On the other hand, although tamoxifen has a relatively narrow therapeutic index and some adverse effects, these are rarely worrying, and the drug has been thoroughly controlled since its approval in 1977 [54]. 

To summarize, the screening of the Prestwick drug library provided a reduced set of promising repurposing drugs for the development of antimicrobial therapies against pneumococcus. Clofilium tosylate, vanoxerine (GBR 12909 dihydrochloride), amiodarone hydrochloride, tamoxifen citrate, terfenadine, and clomiphene citrate impair *S. pneumoniae* growth probably via the interaction and permeabilization of the cell membrane, while the mechanism of the action of mitoxantrone requires future investigation. A better understanding of the corresponding structure–activity relationships could pave the way to obtaining novel pathogen membrane-selective molecules. In any case, as the repurposed drugs are not specifically optimized as antipneumococal agents, further work should also aim to study the synergistic effect of these compounds with current or older antibiotics used in the clinic so as to reinforce their value (threatened by the rise of resistance) while alleviating the pressure on the currently scarce pipelines of new antipneumococcal drugs.

## 4. Materials and Methods

### 4.1. Bacterial Strains and Growth Conditions

The *S. pneumoniae* strains used were R6, a non-capsulated strain derived from the capsular type-II clinical isolate strain D39 [59]; R6CIB17, an R6 spontaneous mutant strain with a non-flocculant phenotype (GenBank accession number: CP038808) [16]; D39 strain [60]; and the ATCC^®^ strain 49619™.

Pneumococcal liquid cultures were grown at 37 °C without aeration in a C medium supplemented with 0.08% (*w*/*v*) yeast extract (C+Y medium) [61]. Growth was monitored by measuring the optical density at 550 nm (OD_550_) in an Evolution 201 spectrophotometer (Thermo Fisher Scientific, Waltham, MA, USA).

### 4.2. Library Screening

The compounds screened in this work correspond to all 1200 off-patent FDA-approved drugs for human use in the Prestwick Chemical Library (Prestwick Chemical, Illkirch-Graffenstaden, France). Stocks were 10 mM in dimethyl sulfoxide (DMSO). All of them were first diluted 20-fold in sterile Milli-Q water, resulting in a drug array at 500 µM in 5% DMSO.

A pre-inoculum (12 mL) of *S. pneumoniae* R6CIB17 was grown until the early exponential phase (OD_550_ of approximately 0.1) and then aliquots of 180 µL were disposed of in a 96-well plate using a fully automated pipette (Rainin E4 XLS+, Mettler Toledo, Columbus, OH, USA) (1.8 × 10^7^ CFU per well). Then, 20 µL of each diluted drug was added to obtain a final volume of 200 µL (final concentration: drug 50 µM in 0.5% DMSO). The plate was introduced in a Multiskan GO (Thermo Fisher Scientific), and the growth curve at 37 °C was monitored using OD_550._ The blanks containing compounds without bacteria were subtracted since some of the compounds showed absorbance at 550 nm. The controls were run in parallel in every plate, containing only bacteria in culture media with and without DMSO 0.5%. All experiments were performed in duplicate.

### 4.3. Susceptibility Tests

The number of viable bacterial cells was determined by counting the colonies from appropriate dilutions of culture (in triplicate) on trypticase soy plates (Conda-Pronadisa) supplemented with 5% defibrinated sheep blood (Thermo Fisher Scientific) after overnight incubation at 37 °C.

The minimal inhibitory concentration (MIC) for each hit was determined in triplicate using the microdilution method, using the Clinical and Laboratory Standards Institute (CLSI) procedure [62]. Briefly, serial 2-fold dilutions of each compound were disposed into 96-well microtiter polystyrene plates (Falcon^®^) containing an R6 pneumococcal cell culture at a concentration of 5 × 10^5^ colony-forming units (CFUs) mL^−1^ in Müller–Hinton media supplemented with defibrinated sheep blood and in a final volume of 100 μL [63]. The MIC was determined as the lowest drug concentration with no visible growth after 24 h of incubation at 37 °C and under 5% (*v*/*v*) CO_2_. Negative controls (bacterial cells in the absence of the tested compound and culture media without bacteria inoculum), as well as a positive control (pneumococcal capsulated strain ATCC^®^ 49619™ in the presence of ampicillin, MIC 0.06 µg mL^−1^), were performed in parallel.

### 4.4. Theoretical Calculations

The calculated value of the LogP parameter (cLogP) was estimated using the Molinspiration web server (https://www.molinspiration.com/cgi-bin/properties, accessed on 17 March 2023).

### 4.5. Cell Permeability Assays

*S. pneumoniae* R6 cells were grown in a C+Y medium at 37 °C up to the final exponential growth phase. Then, they were pelleted via centrifugation (3800× *g*, 4 °C, 10 min) and suspended in half of the starting volume of 5 mM sodium phosphate buffer, pH 7.0, containing 280 mM sorbitol and 1.25 μM SYTOX Green fluorophore (Invitrogen), to a final cell concentration of 2 × 10^8^ CFU mL^−1^. The aliquots of 50 μL of the bacterial suspension were disposed in a 96-well Nunc F96 MicroWell black polystyrene plate (Thermo Fisher Scientific) and read in a Varioskan Flash (Thermo Fisher Scientific) using fluorescence excitation and emission wavelengths of 504 and 524 nm, respectively, to obtain a stable basal signal. Then, 50 µL of each compound (the selected drug stock solution at a final concentration of 25 µM, 1% Triton X-100 for positive control and a buffer for the negative control), previously dissolved in 5 mM sodium phosphate buffer, pH 7.0 plus 280 mM sorbitol, was added to each well, and then the fluorescence monitoring in real time was extended along 95 min. The gain was adjusted using cells incubated with 0.1% Triton X-100 as the maximal value of permeabilization. Experiments were performed in triplicate, and the results were expressed in the percentage of fluorescence intensity relative to the value obtained by the addition of Triton X-100.

## 5. Patents

The results of this work have been submitted to patent protection (Application Reference: WO2022162265A1. Search report: https://tinyurl.com/5f6teszd; accessed on 17 March 2023).

## Figures and Tables

**Table 1:** Antibacterial effects of selected drugs at 25 µM concentration.

Compound	Decrease in OD_550nm_ (%) ^1^	UFC (mL^−1^ × 10^−8^) ^2^	MIC (R6) (µg mL^−1^)	MIC (D39) (µg mL^−1^)	Permeability (%) at 90 min	cLogP
Control (no addition)	-	6.6 ± 0.6	-	-	-	-
DMSO 0.5%	-	4.2 ± 0.3	-	-	-	-
Nortriptyline hydrochloride 25 µM	48 ± 5	6.9 ± 0.2	ND ^3^	ND	ND	3.94
Methyldopa (L, -)	3 ± 1	6.8 ± 0.5	ND	ND	ND	1.23
Pyrvinium pamoate	19 ± 3	5.9 ± 0.3	ND	ND	ND	1.94
Procainamide hydrochloride	31 ± 2	5.7 ± 0.8	ND	ND	ND	0.99
Maprotiline hydrochloride	62 ± 4	5.7 ± 0.5	ND	ND	ND	4.01
Triflupromazine hydrochloride	41 ± 5	5.6 ± 0.8	ND	ND	ND	5.25
Demecarium bromide	46 ± 4	5.3 ± 0.1	ND	ND	ND	−1.42
Chlormezanone	78 ± 9	4.1 ± 0.4	ND	ND	ND	1.44
Flunarizine dihydrochloride	50 ± 3	4.1 ± 0.4	ND	ND	ND	6.09
Metixene hydrochloride	64 ± 7	3.5 ± 0.5	ND	ND	ND	4.68
Methiothepin maleate	58 ± 7	3.3 ± 0.4	ND	ND	ND	4.14
Perhexiline maleate	10 ± 2	3.3 ± 0.3	ND	ND	ND	6.21
Carbetapentane citrate	>99	2.8 ± 0.2	ND	ND	ND	3.83
Bepridil hydrochloride	72 ± 6	2.4 ± 0.2	ND	ND	ND	4.96
Zotepine	58 ± 7	2.3 ± 0.1	ND	ND	ND	5.52
Zuclopenthixol hydrochloride	49 ± 2	1.7 ± 0.6	ND	ND	ND	4.69
Halofantrine hydrochloride	83 ± 8	1.9 ± 0.4	ND	ND	ND	8.55
Clofilium tosylate	>99	0.36 ± 0.02 (9%)	51	38	52	3.34
Vanoxerine	80 ± 8	0.29 ± 0.01 (7%)	26	26	98	6.04
Mitoxantrone dihydrochloride	>99	0.17 ± 0.02 (4%)	>26	>52	<0.1	0.36
Amiodarone hydrochloride	71 ± 9	0.011 ± 0.004 (0.3%)	34	51	71	8.31
Tamoxifen citrate	99 ± 1	<0.001 (<0.02%)	>28	>56	52	6.06
Terfenadine	38 ± 5	<0.001 (<0.02%)	12	47	71	6.17
Clomiphene citrate (Z, E)	91 ± 8	<0.001 (<0.02%)	30	45	64	6.53

^1^ Relative to the control strain R6CIB17 in the presence of DMSO 0.5%. ^2^ Compounds are sorted in decreasing order of viability. Percentages are shown relative to the control strain R6CIB17 in the presence of DMSO 0.5% and only when the value is lower than 10%. ^3^ ND: not determined.

## Data Availability

Not applicable.

## Supplementary Materials

The supporting information can be downloaded at: https://www.mdpi.com/article/10.3390/ijms24065831/s1.
