# Supplementary material for: Drug Repositioning as a Therapeutic Strategy against Streptococcus pneumoniae: Cell Membrane as Potential Target
Source: Int J Mol Sci. 2023 Mar 18;24(6):5831. doi: 10.3390/ijms24065831 (PMC10058218; doi:10.3390/ijms24065831)
Supplement: Supplementary file 1 [file ijms-24-05831-s001.zip › ijms-2264562-supplementary.pdf]

Supplementary information

Drug repurposing/repositioning as a therapeutic strategy against *Streptococcus pneumoniae*: cell membrane as potential target

Laura Ortiz-Miravalles, Manuel Sánchez-Angulo, Jesús M. Sanz and Beatriz Maestro

Figure S1

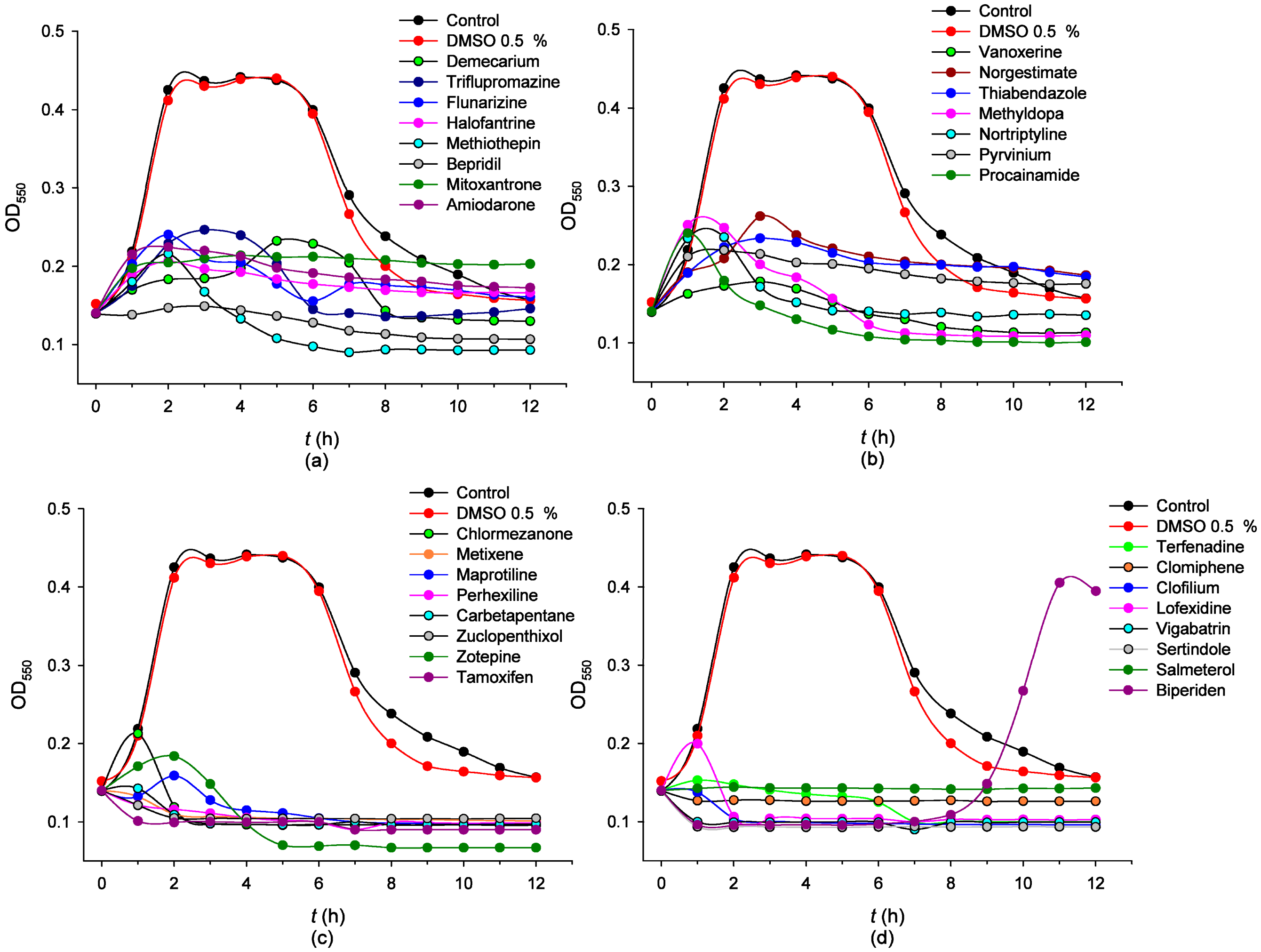

Figure S1. (a-d) Planktonic cultures of *S. pneumoniae* R6CIB17 in the presence of 50 μM concentration of the 31 primary selected molecules (red circles in Figure 1a of the main text). Each compound was added at the early exponential phase.

Table S1

Compound	CAS ID	Chemical structure	Therapeutic approved use	Mechanism of action
1 Thiabendazole	148-79-8	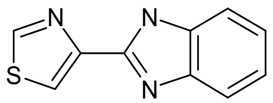	Anthelmintic	Microtubule inhibitor
2 Lofexidine	31036-80-3	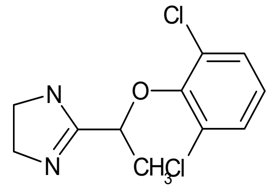	Opinoid withdrawal symptoms, antihypertensive	Alpha2-adrenergic receptor agonist
3 Biperiden hydrochloride	1235-82-1	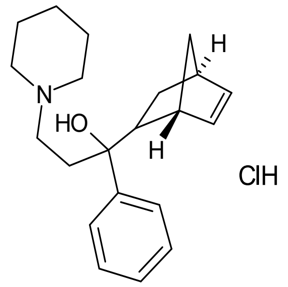	Antiparkinsonian	Anticholinergic
4 Vigabatrin	60643-86-9	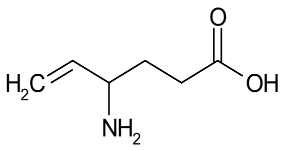	Anticonvulsant GABA	Transaminase inhibitor
5 Norgestimate	35189-28-7	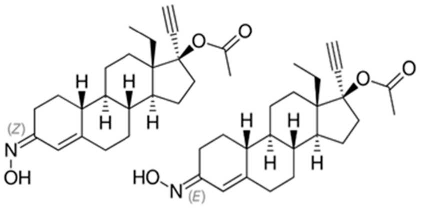	Hormonal contraception Menopausal hormone therapy	Progestogen
6 Salmeterol	89365-50-4	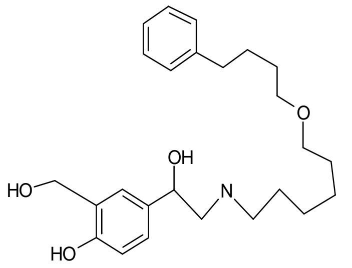	Bronchodilator	Beta 2 adrenergic agonist

Table S1 (cont)

7<br>Sertindole	106516-24-9	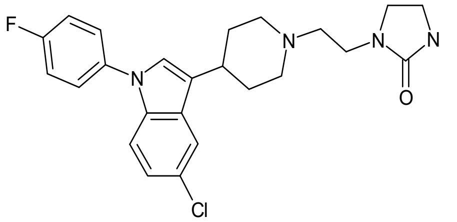	Antipsychotic	Dopamine D2/Serotonin 5-HT2 receptor antagonist
8<br>Nortriptyline hydrochloride	894-71-3	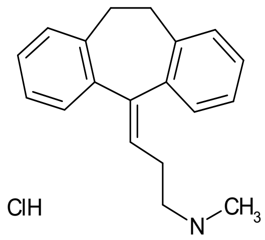	Antidepressant<br>Sedative<br>Analgesic<br>Muscle relaxant	Norepinephrine uptake inhibitor
9<br>Methyldopa (L,-)	555-30-6	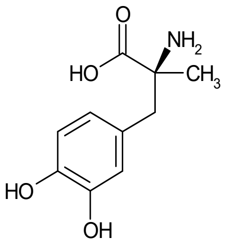	Antihypertensor	L-aromatic aminoacid decarboxylase inhibitor
10<br>Pyrvinium pamoate	3546-41-6	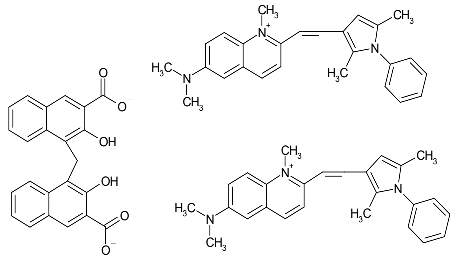	Antiparasitic	Metabolic inhibitor
11<br>Procainamide hydrochloride	614-39-1	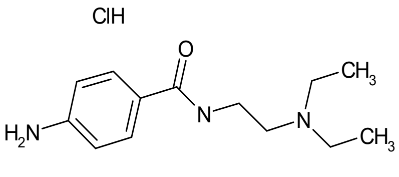	Antiarrhythmic<br><br>Vasodilatator	Alpha antagonist<br><br>Antinuclear antibodies<br>Anticholinergic
12<br>Maprotiline hydrochloride	10347-81-6	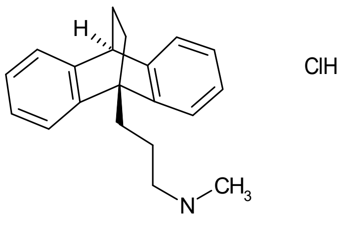	Antidepressant<br><br>Sedative<br><br>Antihistaminic<br>Anxiolytic	Noradrenaline uptake inhibitor<br>5-HT uptake inhibitor
13<br>Triflupromazine hydrochloride	1098-60-8	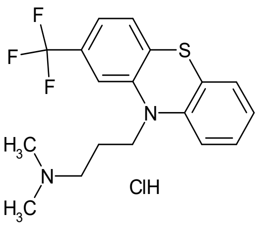	Antipsychotic<br><br>Tranquilizer<br>Antiemetic<br>Antihypertensor	Dopaminergic antagonist

Table S1 (cont)

14<br>Demecarium bromide	56-94-0	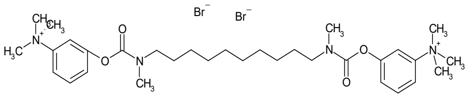	Cholinergic (ophtalmic)	Acetyl-cholinesterase inhibitor (parasympathomime-tic, cholinomimetic drug or cholinergic receptor stimulating agent)
15<br>Chlormezanone	80-77-3	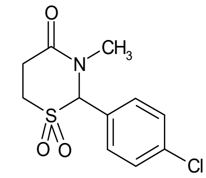	Skeletal muscle relaxant	Potentiator of GABA effects
16<br>Flunarizine dihydrochloride	30484-77-6	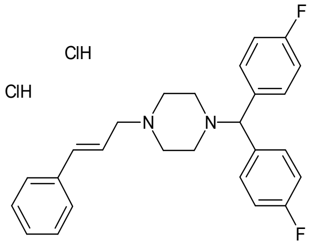	Vasodilatator<br><br>Anticonvulsant<br>Antiarrhythmic	Na^+ channel blocker<br>Ca^{2+} antagonist<br>H1 antagonist
17<br>Metixene hydrochloride	1553-34-0	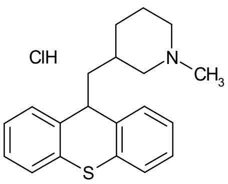	Antiparkinsonian	Anticholinergic
18<br>Methiothepin maleate	19728-88-2	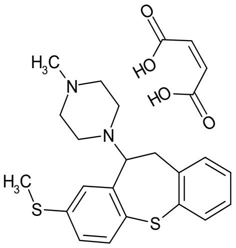	Antipsychotic	5-HT autoreceptor antagonist<br>5-HT1c antagonist<br>5-HT release inhibitor electrical or K^+ induced
19<br>Perhexiline maleate	6724-53-4	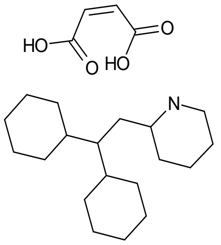	Vasodilatator	Ca^{2+} blocking agent
20<br>Carbetapentane citrate	23142-01-0	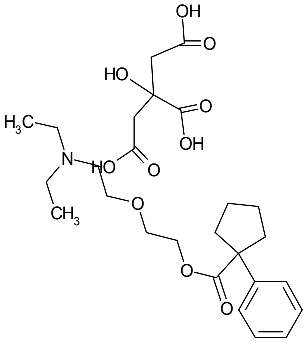	Antitussive<br><br>Spasmolytic	Sigma 1 receptor ligand

Table S1 (cont)

21<br>Bepridil<br>hydrochloride	74764-40-2	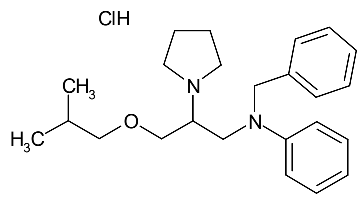	Antianginal	Ca^{2+} channel blocker
22<br>Zotepine	26615-21-4	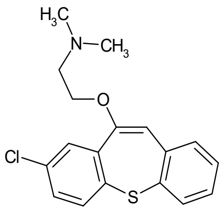	antipsychotic	Dopamine D2/serotonin 5-HT2 antagonist
23<br>Zuclopenthixol<br>hydrochloride	633-59-0	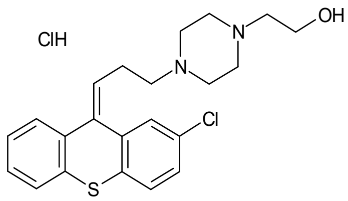	Delirium<br>Schizophrenia<br>Antipsychotic<br>Sedative<br>Neuroleptic<br>Schizophrenia	Dopaminergic receptor antagonist
24<br>Halofantrine<br>hydrochloride	36167-63-2	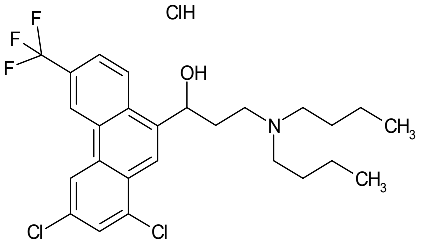	Antimalarial	Blocker of HERG channels
**25**<br>**Clofilium tosylate**	92953-10-1	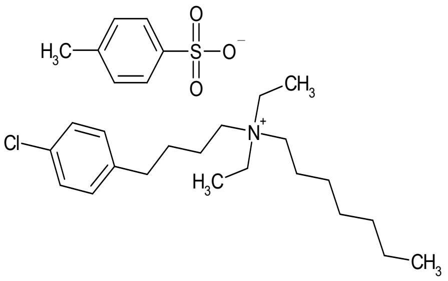	Antiarrhythmic	K^+ channel blocker
**26**<br>**Vanoxerine**<br>**(GBR 12909**<br>**dihydrochloride)**	67469-78-7	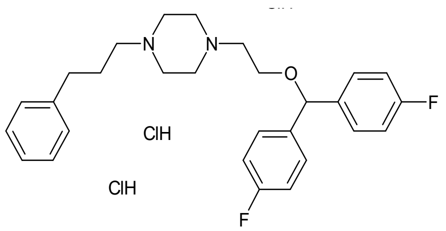	Antidepressant<br>Antiarrhythmic	Dopamine reuptake inhibitor<br>Na^+ channel blocker<br>K^+ channel blocker
**27**<br>**Mitoxantrone**<br>**dihydrochloride**	70476-82-3	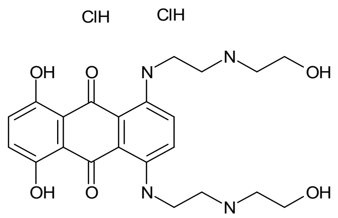	Antineoplastic<br><br>Anticancer	DNA topoisomerase II inhibitor

Table S1 (cont)

**28 Amiodarone hydrochloride**	19774-82-4	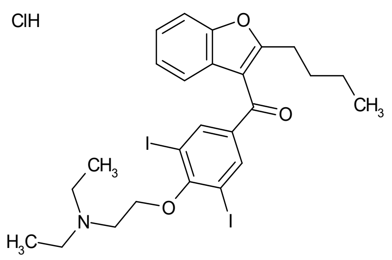	Antiarrhythmic<br>Antianginal	Na^+ and K* channel blocker; Non-competitive beta-adrenergic blocker
**29 Tamoxifen citrate**	54965-24-1	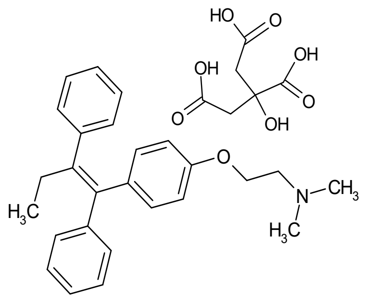	Anticancer	Protein kinase C inhibitor Oestrogen receptor antagonist
**30 Terfenadine**	50679-08-8	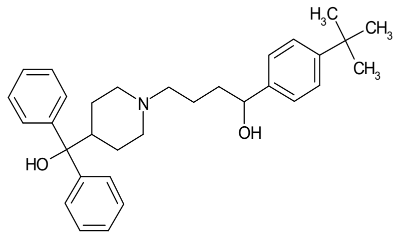	Antihistaminic	H1 antagonist Na^+, K^+ and Ca^{2+} channels blocker
**31 Clomiphene citrate (Z, E)**	50-41-9	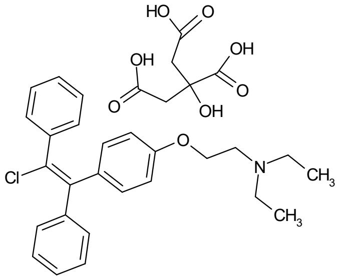	Ovulation inductor	Antioestrogen Gonad-stimulating agent

Table S1. Characteristics of the 31 compounds selected as appreciably affecting planktonic pneumococcal growth at 50 μM and that had not been previously reported as antipneumococcal agents (red circles in Figure 1a of the main text). Those that, additionally, induced at 25 μM a decrease in bacterial viability higher than 90 % relative to the control, are represented in bold (compounds 25-31).
